# Association between weekend catch-up sleep and hypertension of the United States population from 2017 to 2020: a cross-sectional study

**DOI:** 10.3389/fpsyt.2024.1488487

**Published:** 2025-02-04

**Authors:** Yan Luo, Qingyuan Li, Tong Feng, Shasha Meng, Ran Duan

**Affiliations:** ^1^ Respiratory Department, Chengdu Xindu District Second People’s Hospital, Chengdu, China; ^2^ School of Clinical Medicine, Chengdu Medical College, Chengdu, Sichuan, China; ^3^ Respiratory and Critical Care Department, The First Affiliated Hospital of Chengdu Medical College, Chengdu, Sichuan, China; ^4^ The Second School of Clinical Medicine, Southern Medical University, Guangzhou, China; ^5^ Nephrology Department, The First Affiliated Hospital of Chengdu Medical College, Chengdu, Sichuan, China; ^6^ Onology Department, The First Affiliated Hospital of Chengdu Medical College, Chengdu, Sichuan, China

**Keywords:** weekend catch-up sleep, hypertension, sleep patterns, cross-sectional study, NHANES

## Abstract

**Background:**

Hypertension is a prevalent cardiovascular risk factor that significantly contributes to morbidity and mortality worldwide. Previous studies have highlighted the role of inadequate sleep during weekdays in the development of hypertension. However, the potential mitigative effects of weekend catch-up sleep (WCS) on hypertension have been less explored.

**Methods:**

This cross-sectional study analyzed data from the National Health and Nutrition Examination Survey (NHANES) 2017–2020, focusing on American adults. We assessed the association between WCS (defined as the difference in sleep duration between weekend and weekday) and the presence of hypertension. Participants were classified into two groups based on their WCS duration: none (below 1 hours), yes (over 1 hours). Multivariable logistic regression models adjusted for potential confounders such as age, gender, Body mass index (BMI), and lifestyle factors were utilized to explore this association. A Generalized Additive Model (GAM) was employed to generate smooth curves for a nuanced analysis of the nonlinear relationship.

**Results:**

The findings indicated that moderate WCS (less than 4 hours) was significantly associated with a reduced risk of hypertension, while excessive WCS (greater than 4 hours) showed no significant protective benefits.

**Conclusion:**

Moderate weekend catch-up sleep could serve as an effective intervention to mitigate hypertension risk, especially in populations with restricted weekday sleep. These results suggest the potential for integrating sleep management strategies into public health recommendations to address hypertension. Future longitudinal studies are needed to confirm these findings and clarify the mechanisms underlying the relationship between WCS and hypertension risk.

## 1 Introduction

Hypertension, a chronic non-communicable disease, significantly impacts the incidence of cardiovascular diseases, leading to increased healthcare expenditures (1). Globally, the prevalence of hypertension among adults aged 30 to 79 years has doubled from 1990 to 2019 (2). Recognized as a critical public health challenge, hypertension contributes substantially to morbidity from strokes and heart diseases (3). Ongoing research aims to elucidate the pathogenesis and contributing factors of hypertension to enhance preventive and therapeutic strategies. Increasingly, sleep is recognized as an important modifiable risk factor for hypertension, playing a key role in regulating blood pressure (4, 5).

Adequate sleep is crucial for essential metabolic and physiological processes, including the regulation of blood pressure. Historically, research has focused on the relationship between sleep duration and hypertension, indicating that insufficient sleep adversely affects blood pressure (4, 6, 7). Studies suggest that sleeping fewer than the recommended 7 to 8 hours per night increases the risk of hypertension by 36% to 66% (8). Numerous people tend to have reduced sleep hours on weekdays due to time constraints from various obligations. To make up for this sleep deficit, they often engage in longer periods of sleep over the weekend, a phenomenon commonly referred to as “weekend catch-up sleep” (WCS) (9). However, the impact of such variable sleep patterns on hypertension has not been fully determined. Enhancing both sleep duration and quality could be a viable strategy for the management and prevention of hypertension.

There is a noticeable gap in research regarding the relationship between WCS and the risk of hypertension within the American population. Conducting a study with a nationally representative sample of Americans could provide valuable insights and aid in reducing hypertension prevalence. This study utilizes data from a national survey to explore the association between WCS and hypertension among United States population and to examine the dose-response relationship.

## 2 Materials and methods

### 2.1 Study population

**Figure 1:**
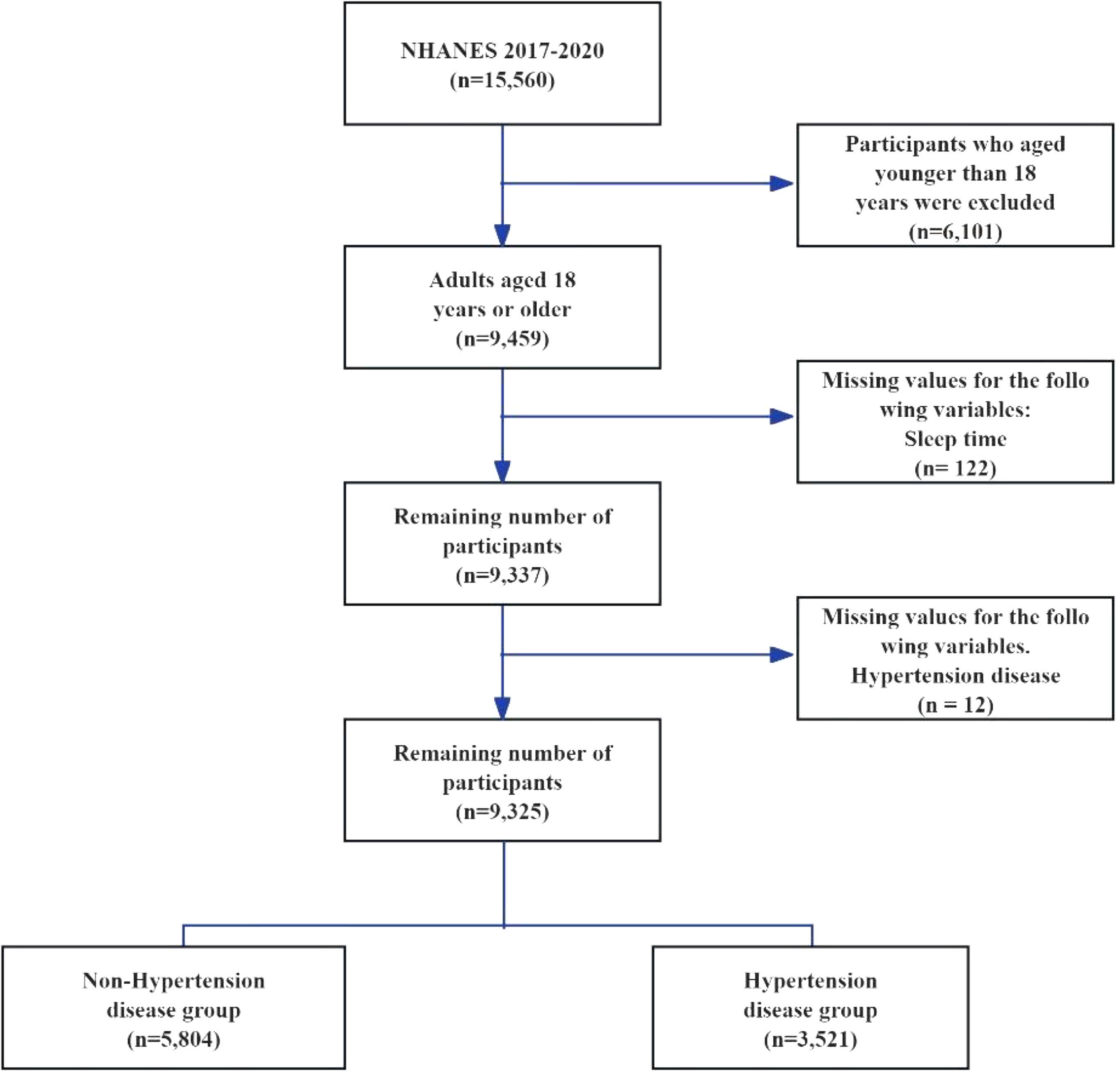
Flow chart for participant selection for the analysis.

This study leverages the publicly accessible he National Health and Nutrition Examination Survey (NHANES) database, which evaluates the health and nutritional status of American adults and children through a comprehensive survey that addresses a variety of populations and health topics. The NHANES database encompasses demographic, dietary, physical examination, laboratory, questionnaire, and restricted access data. Given that previous surveys omitted weekend sleep duration, this research includes data spanning from 2017 to 2020. The study sample comprises individuals aged 18 and older who completed the sleep questionnaire. Initially, the NHANES 2017-2020 cohort contained 15,560 participants. Exclusions were made for those under 18 years old (6,101 participants), those missing sleep duration data (122 participants), and those with no recorded hypertension events (12 participants), resulting in a final sample of 9,325 individuals (**Figure 1**). Of these, 3,521 were diagnosed with hypertension, while 5,804 were identified as normotensive. This study was written in accordance with the STROBE checklist, detailed in **Supplementary Table S1**.

All participants signed informed consent before the survey, and the survey received ethical approval from the Ethics Review Board of the National Center for Health Statistics. The detailed study procedures of NHANES can be found on the NHANES official website (https://www.cdc.gov/nchs/nhanes/). Regarding data usage, this study strictly adhered to the “Data Use Restrictions” regulations.

### 2.2 Assessments of WCS

In the NHANES 2017-2020 survey, the WCS was quantified based on the reported sleep durations of participants on weekdays (Monday through Friday) and weekends (Saturday and Sunday). The questions posed to determine sleep patterns were as follows: During weekdays, participants generally go to bed and wake up at what specific times? How about on weekends – what are the typical sleep and wake-up times for participants during those days? The WCS duration is calculated by subtracting the average sleep duration on weekdays from that on weekends. A normal WCS duration is defined as a difference of 1 hour or less between weekend and weekday sleep durations. If the WCS duration exceeds 1 hour, with weekend sleep exceeding weekday sleep by more than 1 hour, it is classified as significant WCS (10, 11). The specific coding and questions related to sleep issues can be found in **Supplementary Table 2**.

### 2.3 Hypertension definition

Hypertension is typically diagnosed through the administration of antihypertensive medication, a formal medical diagnosis, or the presence of an average systolic blood pressure equal to or greater than 140 mmHg, and/or an average diastolic blood pressure equal to or greater than 90 mmHg (12). The method for determining average blood pressure adheres to specific guidelines: any diastolic readings of zero are omitted from the calculation of the average. In cases where all diastolic readings are zero, the resultant average is recorded as zero. If only a singular blood pressure reading is accessible, that value is utilized as the average. Conversely, when multiple readings are available, the initial reading is disregarded in the calculation of the average.

### 2.4 Covariates

A standardized interview questionnaire was utilized to gather demographic and lifestyle data, including information on age, gender, race/ethnicity (classified as Mexican American/other Hispanic, Non-Hispanic White, Non-Hispanic Black People, and Other Race), marital status, education level (grouped as below high school, high school graduate, and university or higher), family income, smoking habits, and alcohol consumption. Economic status was deduced from family income and poverty levels, with higher income and lower poverty indicating a better economic status. Alcohol consumption in the previous 12 months was evaluated based on the frequency of drinking days and the amount consumed, with categories defined as Never (less than 12 drinks in a lifetime), Former (12 or more drinks in one year but abstinent in the last year), and Current (daily consumption of at least one drink for females and two for males, or binge drinking at least two days per month) (13). Smoking patterns were classified according to current smoking behavior and lifetime cigarette consumption as Never Smokers (less than 100 cigarettes smoked in their lifetime), Former Smokers (over 100 cigarettes smoked in their lifetime but currently non-smokers), and Current Smokers (smoked over 100 cigarettes in their lifetime, either occasionally or daily) (14). Body mass index (BMI) was determined by dividing weight in kilograms by the square of height in meters, with categories established at less than 30 and 30 kg/m² or higher.

### 2.5 Statistical analyses

Participants were then categorized into groups based on hypertension status for descriptive statistical analysis of standard demographic indicators. Continuous variables, whether normally distributed or not, were represented as mean (standard error) and median (interquartile range) respectively. Multiple group comparisons of all continuous variables in this study were conducted using a weighted linear regression model. Categorical variables were presented as frequencies (percentages) and analyzed using the weighted chi-square tests.

A weighted multifactorial logistic regression analysis was performed to investigate the relationship between WCS and hypertension. Model 1 served as a univariate model, Model 2 adjusted for age, gender, and race, while Model 3 additionally adjusted for educational level, marital status, smoking status, alcohol consumption, income-to-poverty ratio, and obesity.

To delve deeper into the potential nonlinear association between WCS and hypertension, a Generalized Additive Model (GAM) was employed to generate smooth curves for a nuanced analysis of the nonlinear relationship within the general population. Furthermore, stratified analyses based on gender, age, BMI, and weekday sleep duration were conducted to assess the impact of these variables on the WCS-hypertension relationship, utilizing likelihood ratio tests to evaluate interactions among stratifying factors.

The statistical analyses were conducted using R software (version 4.1.2, The R Foundation) and EmpowerStats (version 4.1, X&Y Solutions Inc.), with significance considered at a two-sided P-value below 0.05.

## 3 Results

### 3.1 Characteristics of study participants

**Table 1** shows the characteristics of adults with and without hypertension. This study included 9,325 participants, comprising 3,521 individuals with hypertension and 5,894 non-hypertensive controls. The average ages of the hypertension and non-hypertension groups were 58.6 years and 43.4 years, respectively. Males represented 50% of the hypertension group and 47% of the non-hypertension group. The hypertension group had a significantly higher average age (P < 0.0001) but no significant difference in sex distribution (P = 0.0677). Race/ethnicity distributions differed significantly (P < 0.0001), with fewer Mexican American/other Hispanics and more Non-Hispanic Black people in the hypertension group. Marital status varied significantly (P < 0.0001), with more participants in the hypertension group being widowed, divorced, or separated. Educational levels also showed significant differences (P < 0.0001), with a higher proportion of high school graduates in the hypertension group. The income-to-poverty ratio did not differ significantly between groups (P = 0.0756). Smoking behavior differed significantly (P < 0.0001), with more ever/current smokers in the hypertension group. Alcohol consumption was significantly different (P = 0.004), with a higher proportion of never drinkers and ever/current drinkers in the hypertension group. Obesity (BMI ≥ 30.0 kg/m²) was more common in the hypertension group (53.6% vs. 34.3%, P < 0.0001). No significant differences were found in weekday sleep duration (P = 0.2690), weekend sleep duration (P = 0.1911), or WCS duration (P = 0.6367).

**Table 1:** Characteristics of adults with and without hypertension.

Characteristic	Non-Hypertension (N = 5804)	Hypertension (N = 3521)	*P* value
Age (years)	43.4 (42.5,44.4)	58.6 (57.3,59.8)	**<0.0001**
Sex (%)			0.0677
Male	47.0 (44.9,49.1)	50.0 (47.2,52.8)	
Female	53.0 (50.9,55.1)	50.0 (47.2,52.8)	
Race (%)			<0.0001
MexicanAmerican/other Hispanic	18.1 (14.7,22.0)	11.6 (9.2,14.5)	
Non-Hispanic White	62.0 (56.9,66.8)	65.8 (59.7,71.4)	
Non-Hispanic Black	9.5 (7.4,12.2)	13.1 (9.7,17.4)	
Other Race	10.4 (8.3,12.9)	9.6 (7.4,12.3)	
Marital status(%)			<0.0001
Married/living with partner	61.0 (58.3,63.7)	62.8 (59.0,66.5)	
Widowed/divorced/separated	14.2 (13.1,15.4)	26.9 (23.1,30.9)	
Never married	22.8 (20.4,25.4)	9.9 (8.0,12.2)	
Not record	1.9 (1.6,2.3)	0.4 (0.2,0.8)	
Education (%)			<0.0001
Less than high school	10.0 (8.7,11.5)	12.3 (10.8,13.9)	
High school	23.7 (21.0,26.6)	30.3 (26.3,34.6)	
More than high school	64.3 (60.5,67.9)	57.0 (52.9,61.1)	
Not record	2.0 (1.6,2.4)	0.4 (0.2,0.9)	
Income to poverty ratio (%)	3.1 (3.0,3.2)	3.0 (2.9,3.1)	0.0756
Smoking behavior (%)			<0.0001
Never smoker	61.6 (58.6,64.4)	51.9 (49.0,54.7)	
Ever/current smoker	38.4 (35.5,41.4)	48.1 (45.3,51.0)	
Not record	0.0 (0.0,0.1)	0.0 (0.0,0.1)	
Alcohol consumption (%)			0.004
Never drinker	10.09	10.83	
Ever/current drinker	7.9	11.86	
Not record	32.54	27.73	
Obesity (BMI ≥ 30.0 kg/m^2^)			<0.0001
No	64.6 (62.0,67.2)	44.7 (41.2,48.2)	
Yes	34.3 (31.8,37.0)	53.6 (50.1,57.0)	
Not record	1.0 (0.7,1.5)	1.7 (1.2,2.5)	
Weekday sleep duration (hours)	7.5 (7.5,7.6)	7.5 (7.4,7.6)	0.2690
Weekend sleep duration (hours)	8.4 (8.3,8.5)	8.3 (8.2,8.4)	0.1911
WCS duration (hours)	0.9 (0.8,1.0)	0.9 (0.8,0.9)	0.6367

WCS, weekend catch-up sleep; BMI, body mass index.

The bolded P<0.05 represents the statistical significance of the difference between the hypertension group and the non-hypertension group. It indicates that the result is statistically significant, with a P-value of less than 0.05.

### 3.2 Associations between WCS and hypertension

In the primary analysis, individuals who had a weekday sleep duration exceeding one hour compared to weekends were excluded from the study. **Table 2** illustrates the outcomes of logistic regression analyses investigating the relationship between WCS duration and hypertension in three different models. The unadjusted Model 1 shows that the odds ratio (OR) for WCS duration greater than 1 hour versus no WCS is 0.348 (95% CI: 0.281-0.431, P = 0.001). Model 2, adjusting for sex, age, and race, presents an OR of 0.546 (95% CI: 0.415-0.719, P = 0.001). Finally, in Model 3, additional adjustments for educational level, marital status, smoking status, alcohol consumption, income-to-poverty ratio, and obesity were made, resulting in an OR of 0.510 (95% CI: 0.367-0.706, P = 0.002).

**Table 2:** Logistic regression analyses for associations between WCS and hypertension.

Variables	Model 1		Model 2		Model 3	
	OR (95% CI)	*P* value	OR (95% CI)	*P* value	OR (95% CI)	*P* value
WCS (duration > 1h)						
No	Ref.		Ref.		Ref.	
Yes	0.348 (0.281-0.431)	0.001	0.546 (0.415-0.719)	0.001	0.510 (0.367-0.706)	0.002

Model 1: unadjusted.

Model 2: Model 1 + sex, age and race.

Model 3: Model 2 + educational level, marital status, smoking status, alcohol drinking status, income to poverty ratio and obesity.

WCS, weekend catch-up sleep; OR, odds ratio; CI, confidence interval.

### 3.3 Stratified analyses

**Figure 2:**
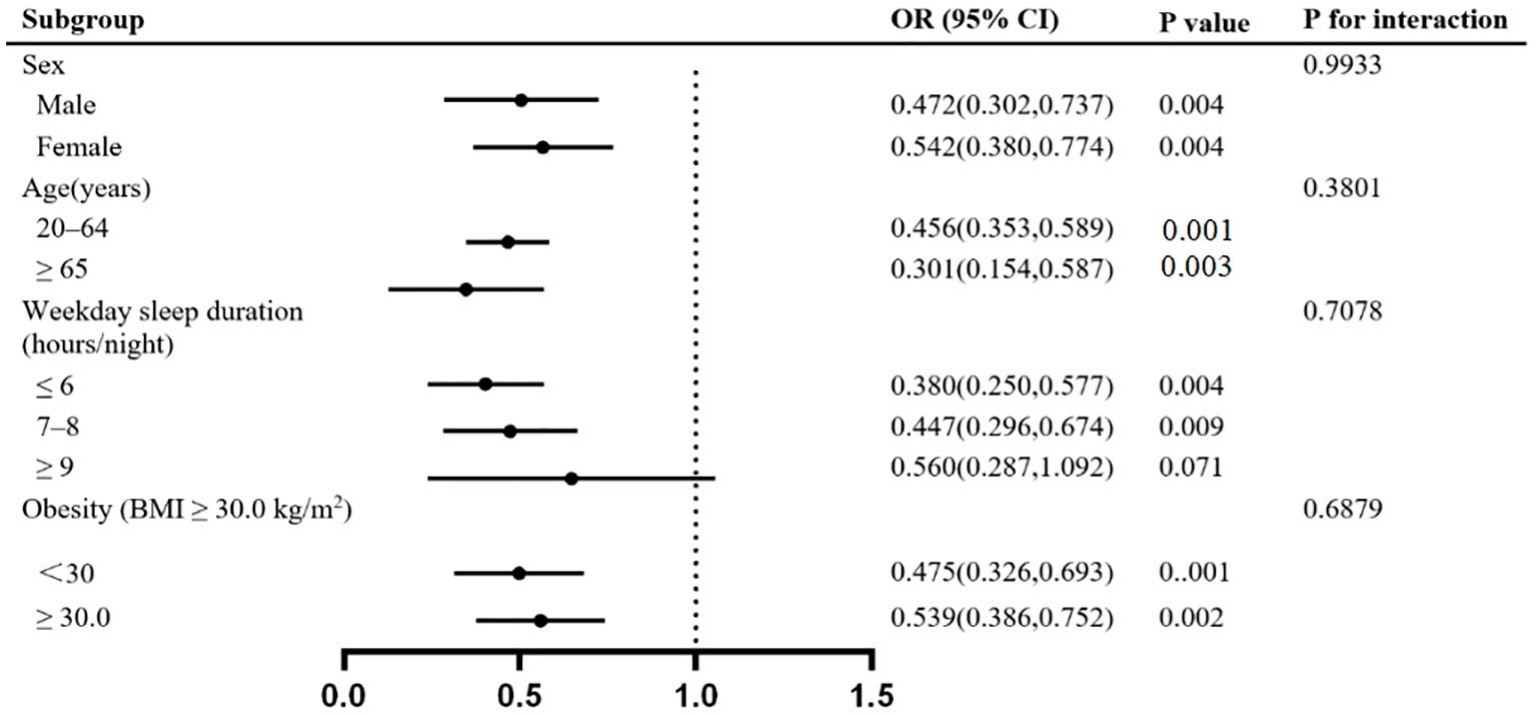
Stratified analyses for the association between WCS and hypertension.

We conducted a stratified analysis based on potential related factors such as gender, age, BMI, and weekday sleep duration (**Figure 2**). The results, shown in **Table 3**, indicate that the association disappears for those with weekday sleep durations greater than 9 hours. No interacting factors were identified.

**Table 3:** Stratified analyses for the association between WCS and hypertension.

Subgroup	WCS (duration > 1h)	OR (95% CI)	P value	P for intercation
Sex				0.9933
Male	No	Ref.		
	Yes	0.472(0.302,0.737)	0.004	
Female	No	Ref.		
	Yes	0.542(0.380,0.774)	0.004	
Age(years)				0.3801
20–64	No	Ref.		
	Yes	0.456(0.353,0.589)	0.001	
≥ 65	No	Ref.		
	Yes	0.301(0.154,0.587)	0.003	
Weekday sleep duration (hours/night)				0.7078
≤ 6	No	Ref.		
	Yes	0.380(0.250,0.577)	0.004	
7–8	No	Ref.		
	Yes	0.447(0.296,0.674)	0.009	
≥ 9	No	Ref.		
	Yes	0.560(0.287,1.092)	0.071	
Obesity (BMI ≥ 30.0 kg/m^2^)				0.6879
<30	No	Ref.		
	Yes	0.475(0.326,0.693)	0.001	
≥ 30.0	No	Ref.		
	Yes	0.539(0.386,0.752)	0.002	

WCS, weekend catch-up sleep; BMI, body mass index; OR, odds ratio; CI, confidence interval.

### 3.4 Dose-response relationship

**Figure 3:**
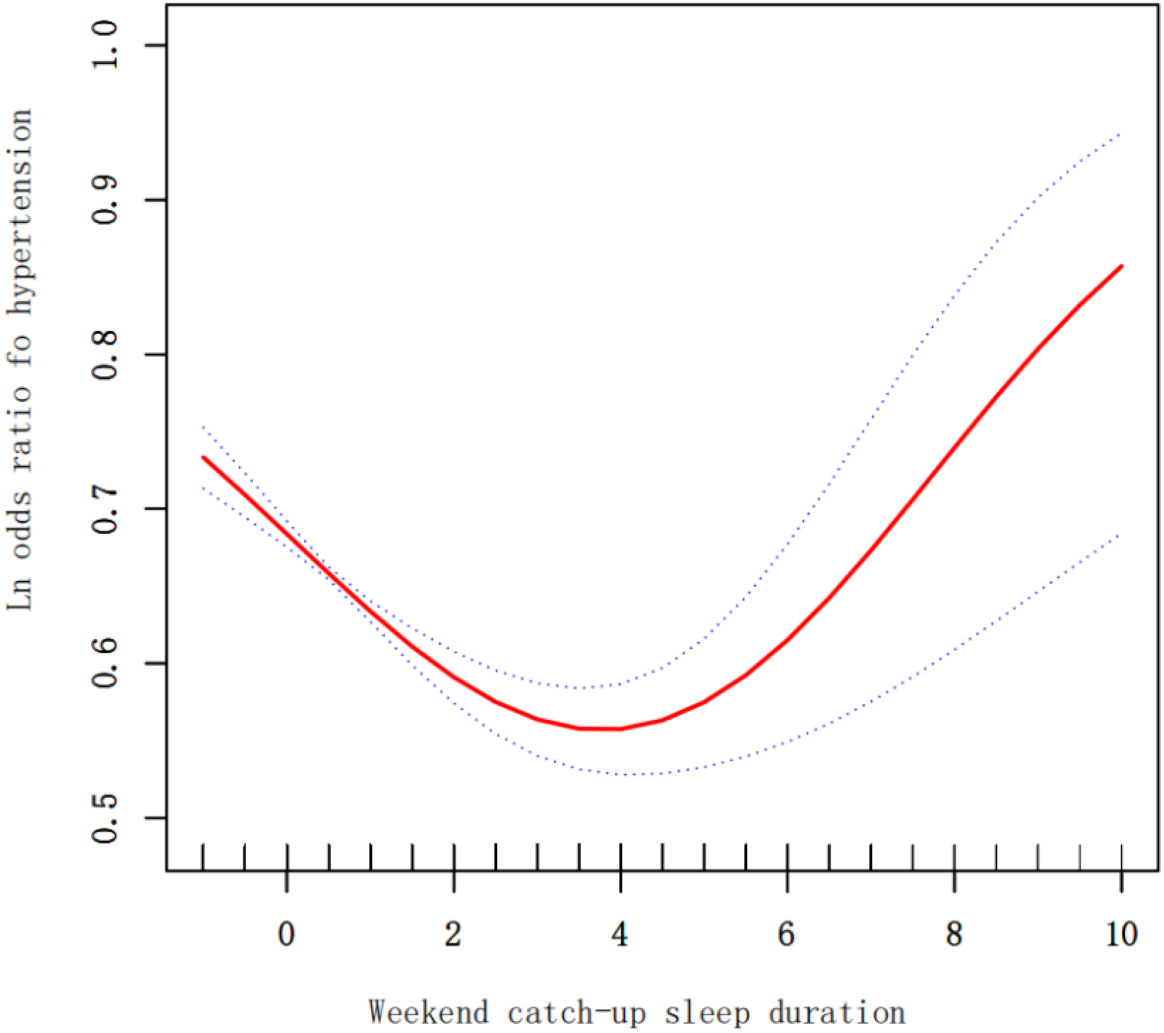
The dose-response relationship of continuous WCS duration with hypertension.

We utilized a GAM to fit a smooth curve and assess the nonlinear relationship between WCS and hypertension in the general population. The results indicated a near U-shaped curve, where the prevalence of hypertension initiall decreased and then increased with higher levels of WCS (**Figure 3**). We conducted a piecewise regression analysis around the inflection point (**Table 4**), revealing that before the inflection point (WCS less than 4 hours), the adjusted OR was 0.86 (95% CI: 0.82-0.90, P < 0.0001), and after the inflection point (WCS greater than 4 hours), the adjusted OR was 1.15 (95% CI: 0.97-1.36, P = 0.1033).

**Table 4:** Threshold effect analysis of WCS and hypertension using the two piecewise regression model.

WCS	Adjusted OR (95% CI), P-value
Total	
Fitting by the standard logistic model	0.90 (0.86, 0.93) <0.0001
Fitting by the two-piecewise logistic model	
Inflection point	4
< 4	0.86 (0.82, 0.90) <0.0001
> 4	1.15 (0.97, 1.36) 0.1033
Log likelihood ratio	0.002

## 4 Discussion

Insufficient evidence exists regarding the potential impact of WCS on hypertension. This study is the first of its kind to explore the connection between WCS and hypertension specifically in American adults. Analysis of data from a comprehensive survey of the American population suggests that WCS is correlated with a decreased likelihood of hypertension among adults. Interestingly, these correlations differ across various subgroups. For adults who get less sleep during the week (≤8 hours per night), WCS is notably associated with a lower prevalence of hypertension. Conversely, in adults who sleep more than 9 hours on weekdays, WCS does not seem to have a significant effect on hypertension risk. These groundbreaking results could offer valuable clinical insights into the intricate relationship between sleep patterns and hypertension. Cardiology practices may benefit from considering targeted approaches to WCS as part of comprehensive hypertension management strategies.

Previous research has also examined the connection between WCS and hypertension. A recent study involving American children discovered that hypertensive children had lower WCS and reported feeling less sleepy compared to the control group (15). The study suggests that increasing sleep duration over the weekend might help counteract the negative effects of sleep deprivation during the week, consistent with our findings. However, the diverse sleep patterns and living situations among young individuals complicate the ability to make direct comparisons with adult populations. Young people often experience varying school schedules, extracurricular commitments, and family dynamics, which can significantly influence their sleep habits. Furthermore, technological engagement such as use of smartphones and computers can also affect their nighttime routines, contributing to irregular sleep patterns. These factors, combined with developmental changes that affect sleep needs and patterns during adolescence, make it difficult to apply findings from adult studies directly to younger demographics. Thus, specialized studies that consider these unique aspects of youth lifestyle and biological development are essential for accurately assessing the impact of sleep on health outcomes in this age group. In the study of adults, WCS duration of more than one hour was significantly associated with a lower likelihood of hypertension compared to those who did not catch up on sleep over the weekend (16). Overall, WCS appears to exhibit protective effect against hypertension in various studies.

This study may be among the first to use GAM to explore the relationship between WCS duration and hypertension. The findings suggest a non-linear correlation between WCS duration and hypertension, indicating that simply sleeping longer over the weekend may not necessarily reduce the risk of hypertension, as previously assumed. These results align with earlier research, particularly studies from the Korea National Health and Nutrition Examination Survey (KNHANES), which showed that only a moderate duration of WCS was significantly linked to improved health outcomes (17–19). This balanced approach considers both the positive and negative effects of WCS duration. On the positive side, WCS can help compensate for sleep deficits accumulated during the weekdays, thereby improving overall sleep quality and reducing the immediate risks associated with sleep deprivation, such as impaired cognitive function and weakened immune response. However, there are also potential negative consequences to consider. For instance, extended sleep durations over the weekend can disrupt the body’s natural circadian rhythm, leading to difficulties in falling asleep and waking up at regular times during the week. This disruption can result in excessive sleep, which has been associated with its own health risks, including increased inflammation and metabolic disturbances (20, 21). Additionally, the phenomenon of social jetlag, where there is a misalignment between an individual’s biological clock and their social schedule, can exacerbate these issues. Social jetlag has been linked to various adverse health outcomes, including an increased risk of hypertension, obesity, and cardiovascular diseases (22). Therefore, while WCS can offer short-term benefits, it is crucial to consider these potential long-term adverse effects when evaluating its overall impact on health. Past studies have highlighted that after a weekend-style recovery sleep, circadian rhythms can experience consistent but moderate shifts, resulting in circadian misalignment and a sense of “jetlag” when individuals revert to their regular weekday schedule. In this study, WCS durations ranging from 0 to 4 hours exhibited more beneficial than detrimental effects, lowering the likelihood of hypertension.

Accumulated epidemiological evidence suggests that WCS has beneficial effects on physical and mental health. However, the underlying mechanisms remain unclear. One possible mediation pathway is through the reduction of inflammation levels. Recent studies have shown that recovering from long-term sleep restriction leads to changes in blood biomarkers, such as myeloperoxidase and its modified low-density lipoproteins, both linked to cardiovascular risk (23). Pejovic et al. conducted a sleep experiment on 30 healthy young individuals and found that plasma IL-6 levels increased during 24 hours of sleep restriction and returned to baseline after resuming sleep (24). Previous research has uncovered a strong correlation between WCS and decreased levels of high-sensitivity C-reactive protein (25). This inverse relationship indicates that individuals who make up for lost sleep on the weekends often exhibit lower levels of systemic inflammation, which is particularly beneficial given hsCRP’s role in assessing cardiovascular risk. Additionally, these findings suggest that proactive sleep strategies like napping or extending sleep duration can enhance the body’s recovery processes linked to immune and inflammatory responses after periods of sleep deprivation. Moreover, WCS may contribute to its positive outcomes by influencing hormone levels, specifically fasting insulin. Studies have shown that just three nights of catch-up sleep can significantly enhance insulin sensitivity in men facing chronic, repetitive sleep limitations. This improvement in insulin sensitivity is crucial as it helps combat the physiological mechanisms that can lead to high blood pressure (26). Notably, insulin has the ability to activate the sympathetic nervous system and boost renal sodium reabsorption, factors known to contribute to hypertension (27). By enhancing insulin sensitivity, WCS may thus play a role in reducing the risks associated with hypertension.

The present study enhances our understanding of the effects of WCS through detailed subgroup analyses. The existing findings on the health implications of WCS, considering age, BMI, and gender, remained consistent. Notably, the subgroup analysis focusing on weekday sleep duration revealed a significant correlation between WCS and hypertension, specifically among adults with shorter weekday sleep durations. This discovery aligns closely with previous studies investigating the relationship between WCS and human health. For instance, Zhu’s research demonstrated the positive effects of WCS on cardiovascular health, particularly among adults who slept six hours or less on weekdays (28). Essentially, individuals with reduced weekday sleep durations may benefit more from compensatory rest. Recognizing the varying degrees of sleep deprivation among individuals with different weekday sleep routines is essential. While our research acknowledges the positive impact of WCS in reducing hypertension, it stresses the significance of attaining adequate sleep throughout the weekdays. Those consistently lacking sleep may struggle to fully repay their sleep debt solely through weekend rest. The build-up of sleep debt during the weekdays can have profound negative effects on cognitive function, mood, and overall health, which cannot be completely offset by weekend recovery alone. Therefore, based on previous research findings, it is advisable for adults to prioritize sufficient sleep both on weekdays and weekends to maintain optimal health. A recent study utilizing data from the UK Biobank revealed a significant correlation between increased sleep regularity and reduced mortality risk, indicating that sleep regularity is a more reliable indicator of mortality risk than sleep duration alone (29). This underscores the importance of maintaining consistent sleep patterns throughout the week rather than relying on erratic sleep schedules. In cases where obtaining adequate weekday sleep is challenging due to demanding work schedules, personal obligations, or other factors, WCS presents itself as a viable alternative worth exploring. While it may not fully offset chronic sleep deprivation, it can still provide some protective benefits by partially mitigating the adverse effects associated with prolonged sleep deficits. Nevertheless, the primary objective should be to cultivate a lifestyle that promotes regular and sufficient nightly sleep to ensure long-term health and well-being.

This study leverages the NHANES database, based on a nationally representative sample of the U.S. population, providing substantial statistical power and enhanced external validity due to its large sample size. The data variables were meticulously collected in a standardized manner, ensuring reliability. By applying appropriate NHANES sample weights and adjusting for a comprehensive array of covariates, the study addresses potential confounding bias, increasing robustness and applicability. The use of generalized logistic and additive models allows for a precise assessment of both linear and nonlinear relationships between WCS and hypertension. Additionally, subgroup analysis and interaction testing ensure that findings are statistically significant and clinically relevant across different population segments.

However, this study has a few limitations. Firstly, as a cross-sectional study, it can only suggest potential causal relationships without establishing causality. These findings, however, provide promising avenues for future longitudinal studies to explore potential causal links. Longitudinal studies would be instrumental in confirming these associations and understanding the temporal dynamics between WCS and hypertension. Secondly, the data on sleep duration was obtained through self-reported questionnaires, which could introduce recall bias. Nonetheless, obtaining sleep duration through objective assessment tools in population-based studies poses challenges, and most epidemiological studies rely on self-reported data. Self-reported measures, while subject to bias, are often the most feasible method for large-scale studies and can still provide valuable insights when interpreted cautiously. Thirdly, shift work is known to induce inflammation and disrupt circadian rhythms, both of which are associated with hypertension development. Unfortunately, shift work status was not collected from participants in the NHANES 2017-2020 cycle, precluding a sensitivity analysis excluding shift workers. The omission of this variable means that we cannot fully account for the effects of shift work on our findings, which could be a confounding factor in the relationship between WCS and hypertension. Despite these limitations, our study provides a comprehensive analysis of the relationship between WCS and hypertension, utilizing a large and diverse dataset, advanced statistical methods, and detailed subgroup analyses to deliver findings that are both robust and clinically meaningful. Lastly, information on sleep apnea or other sleep disorders was only available in the NHANES 2005–2008 cycles and was not included in the 2017–2020 dataset we analyzed. As a result, we were unable to exclude participants with these conditions, which may introduce confounding effects, given the impact of sleep disorders on both sleep patterns and hypertension risk. Future studies should consider controlling for or excluding these variables to enhance the specificity of findings related to sleep patterns and hypertension.

To sum it up, moderate WCS has been shown to lower the risk of hypertension, particularly when practiced for 0-4 hours. This duration appears to provide an optimal balance, offering the benefits of additional rest without the potential drawbacks associated with excessive sleep. The most notable positive outcomes on hypertension were observed in individuals with shorter weekday sleep duration, highlighting the critical role of compensatory sleep for those who are unable to get sufficient rest during the workweek. These findings hold significant implications for public health, suggesting that WCS could be an effective strategy in managing hypertension across diverse populations. Incorporating WCS as a recommended practice could be particularly beneficial for individuals with demanding schedules that limit their ability to achieve adequate sleep during weekdays. Public health campaigns and guidelines could emphasize the importance of not only regular sleep patterns but also the value of compensatory sleep as a practical intervention for those experiencing chronic sleep deprivation. Moreover, understanding the relationship between WCS and hypertension can inform healthcare providers about the potential benefits of advising patients on sleep management strategies. It underscores the need for a holistic approach to sleep health that considers both weekday and weekend sleep patterns. This approach could lead to more personalized recommendations and interventions aimed at reducing the prevalence of hypertension and improving overall cardiovascular health. In conclusion, the practice of moderate WCS emerges as a promising public health strategy, offering a simple yet effective means to mitigate the risk of hypertension. By addressing the sleep deficits accumulated during the week, individuals can potentially improve their health outcomes, making WCS a valuable component of comprehensive health management plans.

## 5 Conclusions

To sum it up, moderate WCS has been shown to lower the risk of hypertension, especially when practiced for less than 4 hours. The most notable positive outcomes on hypertension were seen in individuals with shorter weekday sleep duration. These findings hold significant implications for public health, suggesting that WCS could be an effective strategy in managing hypertension in diverse populations.

## Data Availability

The original contributions presented in the study are included in the article/**Supplementary Material**, further inquiries can be directed to the corresponding author/s.
